# Leptomeningeal Metastasis: A Review of the Pathophysiology, Diagnostic Methodology, and Therapeutic Landscape

**DOI:** 10.3390/curroncol30060442

**Published:** 2023-06-19

**Authors:** Andrew Nguyen, Alexander Nguyen, Oluwaferanmi T. Dada, Persis D. Desai, Jacob C. Ricci, Nikhil B. Godbole, Kevin Pierre, Brandon Lucke-Wold

**Affiliations:** 1 College of Medicine, University of Florida, Gainesville, FL 32610, USA; 2 School of Medicine, Tulane University, New Orleans, LA 70112, USA; 3 Department of Radiology, University of Florida, Gainesville, FL 32610, USA; 4 Department of Neurosurgery, University of Florida, Gainesville, FL 32610, USA

**Keywords:** leptomeningeal disease, metastases, central nervous system, CNS tumor

## Abstract

The present review aimed to establish an understanding of the pathophysiology of leptomeningeal disease as it relates to late-stage development among different cancer types. For our purposes, the focused metastatic malignancies include breast cancer, lung cancer, melanoma, primary central nervous system tumors, and hematologic cancers (lymphoma, leukemia, and multiple myeloma). Of note, our discussion was limited to cancer-specific leptomeningeal metastases secondary to the aforementioned primary cancers. LMD mechanisms secondary to non-cancerous pathologies, such as infection or inflammation of the leptomeningeal layer, were excluded from our scope of review. Furthermore, we intended to characterize general leptomeningeal disease, including the specific anatomical infiltration process/area, CSF dissemination, manifesting clinical symptoms in patients afflicted with the disease, detection mechanisms, imaging modalities, and treatment therapies (both preclinical and clinical). Of these parameters, leptomeningeal disease across different primary cancers shares several features. Pathophysiology regarding the development of CNS involvement within the mentioned cancer subtypes is similar in nature and progression of disease. Consequently, detection of leptomeningeal disease, regardless of cancer type, employs several of the same techniques. Cerebrospinal fluid analysis in combination with varied imaging (CT, MRI, and PET-CT) has been noted in the current literature as the gold standard in the diagnosis of leptomeningeal metastasis. Treatment options for the disease are both varied and currently in development, given the rarity of these cases. Our review details the differences in leptomeningeal disease as they pertain through the lens of several different cancer subtypes in an effort to highlight the current state of targeted therapy, the potential shortcomings in treatment, and the direction of preclinical and clinical treatments in the future. As there is a lack of comprehensive reviews that seek to characterize leptomeningeal metastasis from various solid and hematologic cancers altogether, the authors intended to highlight not only the overlapping mechanisms but also the distinct patterning of disease detection and progression as a means to uniquely treat each metastasis type. The scarcity of LMD cases poses a barrier to more robust evaluations of this pathology. However, as treatments for primary cancers have improved over time, so has the incidence of LMD. The increase in diagnosed cases only represents a small fraction of LMD-afflicted patients. More often than not, LMD is determined upon autopsy. The motivation behind this review stems from the increased capacity to study LMD in spite of scarcity or poor patient prognosis. In vitro analysis of leptomeningeal cancer cells has allowed researchers to approach this disease at the level of cancer subtypes and markers. We ultimately hope to facilitate the clinical translation of LMD research through our discourse.

## 1. Introduction: The Pathophysiology of Leptomeningeal Disease

Leptomeningeal disease (LMD) is commonly characterized as the metastatic involvement of various meningeal regions—the arachnoid mater, subarachnoid space, and pia mater—defined as the leptomeningeal layer. Although LMD can stem from non-cancerous pathologies, such as multiple sclerosis (MS) or meningitis with subsequent inflammation and infection, respectively, this review focused specifically on leptomeningeal metastasis secondary to a neoplasm or carcinomatosis. Prevalence exists in 1–8% of all cancer patients, with a recorded incidence of 110,000 cases in the US annually [1]. Furthermore, the prevailing sites of origin include breast and lung cancer, melanoma, and lastly, primary central nervous system (CNS) cancers [1]. Additionally, hematologic origins, namely non-Hodgkin’s lymphoma (NHL), acute lymphoblastic leukemia (ALL), and multiple myeloma, are seldom negligible in occurrence [2]. Several mechanisms for the general onset of LMD have been purported: (1) direct invasion via surrounding structures, such as the dura mater, bone, or nerves; (2) hematogenous spread often by way of venous vasculature; and, lastly, (3) entry of the fenestrated pores of the choroid plexus typically permitting solute transport [1,3]. Within LMD-related cerebrospinal fluid (CSF), the presence of increased levels of complement component three protein (C3) has been observed. Postulations describe the role of C3 in its ability to interact with choroid plexus C3a receptors, thereby increasing the endothelial permeability of the normally intact barrier [4].

Clinically, the presentation of LMD includes non-specific, generalized neurological symptoms, such as headaches, confusion, seizures, and radiculopathy, among others. Though this substantially shifts the weight of efficacy to other modes of diagnosis, more refined schemas for focused diagnosis have been formally developed. For example, within cerebral localizations, symptoms may manifest as headache and confusion, while cranial nerve (CN) deficits may be attributed to involvement of the posterior fossa; the anatomical afflictions and their corresponding presentations are described more extensively in recent literature [3]. Regarding prognosis, the National Comprehensive Cancer Network (NCNN) has established technical criteria for stratification of prognosis [5]. Karnofsky Performance Scale (KPS) values < 60, systematic neurological presentation, and encephalopathy have all been associated with a higher risk of disease progression. Additionally, hematologic origins have displayed seemingly better outcomes [6]. To date, the clinical repertoire for guiding differential diagnoses has been through obtaining medical history and physical examination. It is important to note the overlap between COVID-19 neurological symptoms and findings with leptomeningeal metastasis. The two pathologies share both molecular and structural shifts. Namely, COVID-19 patients have been shown to present with CSF positive for LMD inflammatory cytokines and MRI involvement within LMD regions of interest. Similarities are also found in other disorders, such as Sturge-Weber syndrome (SWS), due to the presence of leptomeningeal angiomatosis. Overall, the median time from diagnosis of primary cancer to LMD has ranged from 1.2–2 years for solid tumors and 11 months for hematologic cancers [3].

**Figure 1:**
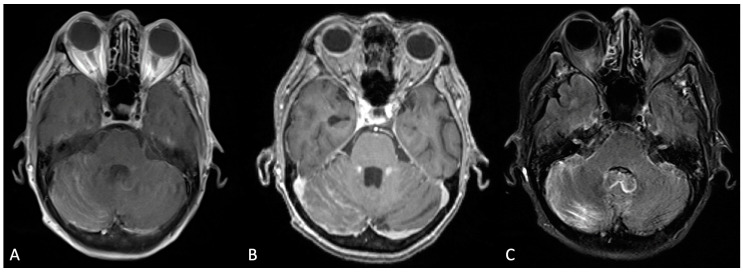
Bilateral cerebellar folia hyperenhancement, more prominent on the right, was observed in the post-contrast T1-weighted Turbo Spin Echo (TSE) sequence (**A**); the T1-weighted Multi-Planar Reconstruction (MPR) sequence (**B**); and the T2-weighted Turbo Inversion Recovery Magnitude Fat Suppressed (TIRM-FS) sequence (**C**).

On the matter of modality-based diagnosis, the gold standard comprises two modalities in the form of imaging and cytological analyses. T1-weighted magnetic resonance imaging (MRI) with gadolinium contrast is the imaging tool of choice. The observational patterns to hearken to include various morphologic enhancements of the cranial nerve, or linear/curvilinear enhancements, and nodular aberrancies [2,7]. These are often noted in select areas, such as the cerebral convexities, basal cisterns, and ventricular ependyma [4]. Figure 1 depicts an MRI scan of leptomeningeal involvement following breast cancer metastases. Within the spine, particularly in the cauda equina region, similar observations should raise suspicion for LMD [4]. Preliminary MRI is becoming an increasingly routine measure for early brain metastasis. As such, several structure densities along the brainstem, cranial nerves, meninges, and ventricles have been correlated with leptomeningeal metastasis. Of note, some findings, such as increased ventricle MRI density, can be a consequence of LMD-associated disorders, in this case hydrocephalus. Since negative MRI findings are not exclusionary, the second modality, CSF cytology, is an essential layer in the diagnosis of this pathology. Conventional cytology hinges on the presence of malignant cells, irrespective of their primary origin, as determined by a cytopathologist. However, cancer specific markers may aid in the determination of the origin, such as the presence of VEGF. In general, the sensitivity and specificity of LMD diagnosis following CSF cytology range from 50–60% and 75–80%, respectively, upon first and second aspirations of CSF [1,3]. Therefore, it is highly encouraged to conduct a second analysis of the CSF when possible, in order to increase the diagnostic capacity for LMD. As a soft requirement, a minimum of 10 CSF mL should be collected for sufficient analysis, with some studies reporting a minimum of 5–10 mL [1,3]. Additionally, it is recommended that the suspected region of affliction (based on several parameters, such as clinical presentation and imaging) be the area of aspiration; in cases where this is not feasible, the lumbar or cisternal regions should be considered as immediate alternatives [1]. The select biomarkers that are measured to supplement ruling in LMD include pleocytosis, hypoglycorrhachia, and hyperproteinorrachia (elevated CSF protein levels). These findings have been observed with a sensitivity of 50–70% [8]. More novel forms of investigation have arisen in the form of tumor-specific antigens, such as carcinoembryonic antigen (CEA) and alpha-fetoprotein. The latest of these endeavors has manifested itself by relying on cell-free DNA as a source of direction [3]. The sensitivity of this latter technique has been nascently explored, though its sensitivity has been reported at 94% with a specificity of 100% [9]. Indeed, in a recent meta-analysis of 668 patients with circulating tumor cells (CTC) and cell-free tumor DNA, sensitivities and specificities were reported at 87.0% and 97.9% (sensitivity, respectively) and 93.8% and 89.0% (specificity, respectively). This has been more robustly assessed in particular cancer-specific LMDs, such as breast cancer [10]. This includes optimization of isolating and purifying circulating tumor-specific DNA and subsequently identifying targetable mutations. Abnormal MRI and CSF findings, as previously mentioned, in the presence of generalized neurological symptoms can be cause for LMD suspicion.

Currently, treatment for LMD secondary to metastasis offers conventional cancer treatment in the forms of surgery, radiotherapy, and chemotherapy. Surgical intervention primarily involves the installation of interventricular delivery devices (i.e., the Ommaya reservoir) or management of LMD complications, such as hydrocephalus and addressing excessive intracranial pressure (ICP); this entails a ventriculoperitoneal shunt (VPS)-dependent manner of draining excess CSF [11]. Radiation is typically indicated for LMD with the sole aim of palliation due to the late onset of diagnosis, particularly in the form of multiple brain metastases. This is implemented in the form of whole brain radiation therapy (WBRT) for cranial afflictions, with spinal localization utilizing traditional fractionated radiation. Radiation involvement is typically forgone lest there be symptomatic involvement or bulky disease [5]. Recent explorations have begun to assess the efficacy of more invasive measures, namely, craniospinal irradiation (CSI). Its adequacy has been particularly salient in cancers of hematologic origin, encouraging timely invitations for extrapolated interest in solid cancers [12]. Dosing ranges have been reported at 20 to 40 Gy in 5 to 20 fractions, marking minimum and maximum cumulative doses of 400 Gy and 800 Gy, respectively [11].

Immunotherapy is delivered based on either the intention of systemic engagement or focused, targeted therapies. Systemic immunotherapy, widely studied across various cancers and metastatic diseases, includes immune checkpoint inhibitors (ICIs) to pragmatically modulate the endogenous immune system. Currently, high-level evidence in the form of randomized controlled trials (RCTs) assessing systemic immunotherapies in LMDs remains scarce. The dearth is occupied by the limited validity of various case reports, with a single RCT displaying limited potential for the four included LMD patients [13]. The proceeding sections will discuss primary cancer-specific therapeutics, with a major focus on targeted immunotherapies [14,15,16].

Though the overarching mechanisms for the pathophysiology of LMD have been discussed, it is well acknowledged that the mode of origin precluding the metastases carries significant implications for engaging this disease. This manifests in the form of diverse biomarkers, treatments, and epidemiologic profiles. Such variation may influence the capacity and manner of treatment for the particular form of LMD. Therefore, the aim of this review is to coalesce the available literature on LMD across the various primary origins, particularly the most prevalent forms. Furthermore, it will cohesively synthesize the current treatments, both preclinical and clinical, while highlighting similarities and differences.

## 2. Breast Cancer

Breast cancer remains one of the leading cancer types by diagnosis, and cases have been steadily rising over the past decades [17]. While meningeal carcinomatosis (MC) is a rare metastatic outcome occurring in roughly 1–5% of solid tumors, primary breast tumors, along with lung tumors and melanoma, make up the most common origin sites [18]. Among studies of patients with leptomeningeal breast cancer metastasis (LBM), the average age of breast cancer diagnosis is often between 50 and 60 [18,19,20,21,22,23]. Once diagnosed, the average time to develop LBM is less than 10 years [19,23]. Unfortunately, survival after leptomeningeal metastasis remains poor. Over multiple reviews and meta-analyses, the average survival time ranges from 16 weeks to 2 years [18,19,20,24,25,26,27].

Invasive breast cancer can be broken down into two common types: ductal and lobular carcinoma. These can be further broken down into four subtypes: luminal A, luminal B, HER2, and triple-negative cancer [28]. Luminal A cancers are either estrogen receptor (ER) or progesterone receptor (PR) positive but human epidermal growth factor receptor 2 (HER2) negative. Luminal B cancers are ER-positive, PR-positive or negative, and HER2 negative. They are characterized by levels of Ki67 expression over 20%, equating to faster growth relative to luminal A tumors. HER2 cancers are HER2 positive and ER/PR negative. These tumors have been the focus of research for many years due to their faster growth than luminal tumors and poorer prognosis. However, since the introduction of therapies that specifically target the HER2 protein, overall survival has improved. Finally, triple-negative breast cancer is ER/PR/HER2-negative and is notorious for presenting in later stages. It is characterized by its aggressiveness and propensity for relapse [28].

In relation to the pathogenicity of LBM, there has been a heavy focus on the role HER2 plays, though the overall pathogenesis remains to be elucidated. HER2 is similar to the epidermal growth factor receptor (EGFR) and is a transmembrane protein that has tyrosine kinase activity once activated by interactions with HER3 or EGFR [29]. Multiple studies have implicated these interactions in the development of MC. Among these, it has been shown that the dimerization of HER2 and HER3, as well as levels of Src, an enzyme involved in the HER2-HER3 dimerization process, lead to increased extravasation and increased blood-brain barrier (BBB) infiltration [30,31]. Interestingly, HER2 status does not determine the possibility of MC development, as studies have shown that around 20% of CNS metastases from HER2- breast primary tumors show cells that are HER2+ [32].

Given that MC is a rare complication, it is often observed later in the disease course in patients who have already developed metastatic lesions. It has been purported that established brain metastases carry an increased risk of developing MC [33,34]. Other risk factors include age at diagnosis and metastasis at other extracranial sites [35]. One study has shown that surgical resection of brain metastases increases the risk of developing MC [33]. In patients who develop MC without other CNS lesions, studies have shown that there is significantly better overall survival as compared to those patients who develop MC with brain metastases as well as patients with brain metastases alone [20].

**Figure 2:**
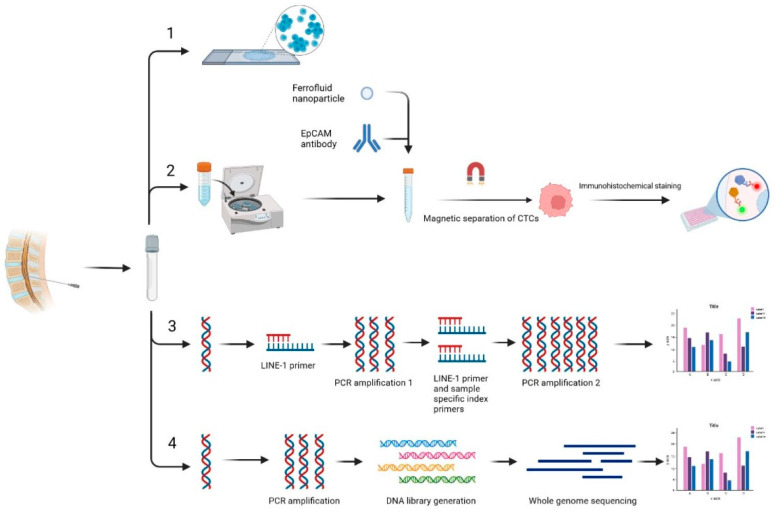
Schematic of current methods for diagnosing meningeal carcinomatosis. All methods begin with CSF sample collection. The current gold standard is CSF cytology (1). (2) describes the process of identifying tumor cells in CSF samples using CellSearch technology. (3) is the method of identifying cell-free DNA (cfDNA) using mFAST-SeqS. (4) shows the method of identifying cfDNA using ultra-low-pass whole genome sequencing (ulpWGS).

As MC has a poor prognosis, it is imperative to diagnose it early and provide the patient with the best possible opportunity to respond to treatment and prolong their life. Unfortunately, the gold standard for diagnosis of MC is MRI and CSF cytology, which carry a low sensitivity in diagnosing MC in its early stages [36,37,38]. As such, there has been a push to develop more sophisticated diagnostic tests to detect MC. One method has utilized CellSearch cell capture technology to detect circulating tumor cells in the CSF of patients with suspected MC (Figure 2) [39]. Though their sample size was small, they noted the ability of this technology to detect circulating tumor cells in CSF and mentioned one patient with negative CSF cytology but positive circulating tumor cells who subsequently developed positive cytology [39]. A subsequent study described a method that utilized next-generation sequencing and fast aneuploidy screening test sequencing to determine the presence of tumor-derived DNA in the CSF of patients with MC. They found that the method was successful in 93% of samples, and MC was diagnosed in 75% of samples in which aneuploidy was detected by this method. Notably, a third (8/24) of samples in which aneuploidy was detected were concurrently diagnosed with MC or developed it soon after this study was conducted [40]. Finally, a study published in 2022 assessed the feasibility of using ultra-low pass whole genome sequencing to detect tumor-derived DNA (ctDNA) in CSF. The benefit of this method is that it does not require the sequencing of a primary tumor. While they found ctDNA in all patients with diagnosed MC, they showed that greater suppression of ctDNA levels during treatment was associated with longer overall survival, and an increase in ctDNA levels was detectable 12 weeks prior to clinical progression of the disease [41]. With respect to imaging detection, Pan et al. detailed a case report in which an LBM patient demonstrated a “hot cross bun” (HCB) shape indicative of multi-region brain deterioration. Although these T-2 MRI hyperintensities overlap with other neurodegenerative diseases, the patterns may be a beneficial tool in monitoring the status of individuals with breast cancer. Overall, there are multiple methods that show promise for improving the ability to detect MC, but robust trials are still necessary.

Treatment strategies for MC include intrathecal chemotherapy, systemic chemotherapy, and radiotherapy. While recent advances have shown better control of the disease, there is still a lack of robust randomized control trials, and substantial improvements in overall survival have yet to be shown. Given this, there has been a focus on prognostic factors to help manage patient care. By far, the most commonly reported predictor of poor prognosis is performance status [22,23,42,43]. However, studies have also exhibited that negative prognostic factors include negative hormone receptor status, higher histological tumor grades (2 and 3), and the use of more than three treatment lines [22,23,42,43,44]. The use of concomitant therapy has been shown to be a positive prognostic factor [44]. There have been multiple clinical trials analyzing treatment strategies for MC. Recent Phase II trials have shown that intrathecal liposomal cytarabine combined with systemic chemotherapy improves overall survival by roughly 3 months [45]. For patients with HER2-positive cancers, a study has shown that intrathecal administration of trastuzumab prevented neurological progression-free survival in 75% of patients at 10 weeks with no significant toxicity [46]. Lastly, in patients receiving radiotherapy, a study comparing proton and photon irradiation showed that proton irradiation was associated with longer CNS progression-free survival and longer overall survival compared to photon therapy. Currently, there is a clinical trial enrolling patients that is investigating the combination of pembrolizumab and Lenvatinib for controlling MC (NCT04729348). While these studies have shown promising results, it should be kept in mind that trial populations are small as it is a rare complication, and larger trials are necessary to determine if effects are of statistical and clinical significance.

## 3. Lung Cancer

Albeit rare, leptomeningeal lung cancer metastasis (LLM) can occur, including non-small cell lung cancer (NSCLC), which includes adenocarcinomas, squamous cell carcinomas, and large cell carcinoma [47]. Lung cancer is the leading cause of cancer death in the United States, with approximately 57% of NSCLC patients progressing to a metastatic state [48]. The CNS is a common site for lung cancer metastases, with 10–20% of patients with NSCLC exhibiting brain metastases (BM) at the time of diagnosis [49]. From the start of intracranial involvement, 2–12% of cases exhibit LMD, although LLM can present later in patients as well, even up to several years following diagnosis [50,51]. In individuals 40 years of age or younger, the incidence of lung cancer remains relatively low and rises significantly from ages 65 to 84 [47]. The age of patients with a solid LMD malignancy is consistent in multiple studies, with most reports showing patient age being in the late 50s [52,53]. Certain demographics of patients with NSCLC can be at increased risk of intracranial dissemination as well. There is strong evidence from many studies that patients with an advanced, local EGFR protein mutation present in an adenocarcinoma are at higher risk of developing BM. Almost universally, studies have shown that women have a higher frequency of EGFR mutations than men, with up to 42% of females with NSCLC exhibiting mutations and male percentages being up to 14% [54,55]. Patient cases of anaplastic lymphoma kinase (ALK)-rearranged NSCLC show a similar increase in rates of BM [56]. LLM following NSCLC has a very poor prognosis. Even with aggressive treatment and advances in therapeutic approaches, survival remains low at 3 to 11 months following diagnosis and treatment [57]. Advantageously, LLM from NSCLC is more readily detected due to recent improvements in imaging and treatment of NSCLC. Small-cell lung carcinoma (SCLC) may also lead to LLM, with the incidence estimated to be 10% in patients with solid tumors [58].

The most common type of NSCLC is lung adenocarcinoma (LUAD), which occurs in 47% of cases in Western countries. After LUAD, lung squamous cell carcinoma (LUSC) is the second most common. Tobacco smoking is the main risk factor for developing lung cancer, with growing evidence that up to 90% of lung cancer cases are significantly caused by active or passive smoking [59]. In addition, air pollution is responsible for approximately 5% of lung cancer-related deaths [60]. The development of NSCLC and SCLC is also influenced by factors like age, BMI, chronic disease, diet, and alcohol consumption. LLM develops through hematogenous spread, endoneurial or perineural routes, and direct seeding through metastases in the brain or cranium [61]. As mentioned before, lung cancers have the potential to be highly metastatic, with up to 50% of cases being metastatic at diagnosis and a common target being the brain [62,63]. In metastasis, cancer cells engage in a cascade consisting of local tissue invasion, intravasation into the blood, circulation, extravasation, and colonization in the target organ. As previously noted, BMs are seen increasingly in LUADs with EGFR mutations and ALK rearrangements. Many factors regulate tumor cell migration, growth, and invasion, including cytokines, adhesion molecules, and gene activity. One example was the increased level of metastasis and subsequent poor prognosis found in LUADs that exhibited high smooth muscle actin gene (ACTA2) activity [64]. It is important to note that there is likely no exact formula of necessary gene activity for each lung tumor cell to reach metastasis; it is more likely that each lung tumor cell has adapted to its circumstances and developed its own mechanism to reach a metastatic state, as the migration of tumor cells is regulated by different genes [64].

Patients with LMD can present with a wide variety of symptoms due to different areas of the CNS being affected. The most common presentations of LMD include headache or back pain, nausea, vomiting, cranial nerve paralysis, walking difficulties, seizures, and neurological defects [65]. Even so, these symptoms are mostly nonspecific and cannot alone justify a diagnosis of LMD. Extensive clinical testing and thorough neurological evaluations need to be made before a diagnosis can be made [65]. The EANO-ESMO guidelines for LMD diagnosis and treatment were created in 2017 and provide specific diagnostic criteria and neuroimaging- and cytopathology-based classifications of LM syndromes to derive pragmatic treatment algorithms. Many details of the epidemiology, prognosis, and therapeutics are detailed in the 2017 study. The gold standard for comprehensive LLM patient imaging is cerebrospinal gadolinium-enhanced MRI of the brain and spine [65,66]. Classic imaging abnormalities on brain MRI are diffuse leptomeningeal enhancements along the gyri, sulci, cortical surfaces, cranial nerves, and cerebellar folia [67]. A CSF biopsy is a necessary follow-up to confirm the presence of LMD, with the gold standard being CSF cytology. Epithelial cancers, such as lung cancer, express epithelial cell adhesion molecules (EpCAMs), which can be a marker used in EpCAM-based flow cytometry, a method of LMD diagnosis that may have a higher sensitivity and specificity than CSF cytology [65]. Recently, there have been efforts to find novel biomarkers that can point to LLM. One 2023 study shows that specific CSF exosomal microRNAs can be potential biomarkers for NSCLC LLM, providing several examples [68].

There are currently several ongoing studies on proposed novel CNS therapies and targets for NSCLC. CHRYSALIS is an ongoing study seeking to evaluate the use of Amivantamab (an IV-administered monoclonal antibody targeting EGFR and MET mutations) and Iazertinib (an orally administered EGFR tyrosine kinase inhibitor (TKI)) in patients who have not received treatment and in relapsed patients [69]. MARIPOSA is a clinical trial that seeks to compare the safety and efficacy of the combination treatment found in CHRYSALIS to single-agent Osimertinib for EGFR-mutated NSCLC [70]. TRIDENT-1 is a study evaluating the use of repotrectinib (a ROS-1 inhibitor/TKI in ROS-1) in ROS-1 mutant NSCLC patients [69]. Early trials in the study show that the treatment is well tolerated [69].

Treatment of LMD mostly focuses on symptom alleviation and palliation due to the poor prognosis of the condition. Radiotherapy is one of the most common approaches to all types of LMD management and has been widely used. Craniospinal irradiation, which administers radiation to the entire neuroaxis, has recently been utilized with modern-day techniques. One such technique, proton beam therapy, has been emphasized as having a lowered risk of treatment toxicity [71]. Systemic chemotherapy in combination with intrathecal chemotherapy remains the standard treatment for non-nodular types of LM. As systemic chemotherapy has been reported to be an independent predictor of survival, it is the treatment of choice for NSCLC patients with LLM [57]. A consensus has not been reached on the standard of chemotherapy, although bevacizumab-erlotinib and pemetrexed have been used successfully in previous clinical trials [57]. Intrathecal chemotherapy shows a significant level of efficacy for NSCLC patients, but the optimal drug of choice, schedule, and dosing regimen have not yet been established. Methotrexate, cytarabine, and thiotepa remain the three most commonly administered intrathecal chemotherapy drugs, although intrathecal methotrexate administered alone remains the most widely utilized and the most efficacious [72]. Molecular targeted therapy should be considered when treating NSCLC patients with LLM, as it has a history of improving clinical outcomes. Patients with EGFR mutations may benefit from EGFR tyrosine kinase inhibitors. Gefitinib, a first-generation EGFR inhibitor, has been shown to increase favorable outcomes among LUAD patients with LMD when given at standard or high doses [57]. Afatinib, Osimertinib, and AZD3759 are tyrosine kinase inhibitors that have shown significant cranial and CNS penetration [57].

## 4. Melanoma

Melanoma has the highest association with LMD, and approximately 5% of melanoma cases metastasize to the leptomeninges. The incidence of leptomeningeal melanoma metastasis (LMM) is between 5–25% and can be as high as 30% [3,73]. This incidence is undervalued because undiagnosed LMD is commonly found post-mortem [73]. In a retrospective review by Chorti et al. (*n* = 52), the median time between the diagnosis of primary melanoma and LMM was 8.5 months [74]. LMM can occur at any age, and while the risk of melanoma increases as we age, on average, patients are diagnosed with melanoma at the age of 65 [75]. The prognosis for patients with LMM remains grim at 17–22 weeks despite advancements in treatment [73,74,76]. There are exceptions to this survival duration range, as exemplified by a case report from Marinova et al., demonstrating that a 43-year-old patient with BRAF-positive stage IIIa melanoma was responsive to radiation and immunotherapy. The patient continued to survive for 2.5 years with neurological symptoms [76]. Many patients with LMM fail to respond to treatment and often die from severe systemic disease burden, tumor progression, and medication toxicity. It has been theorized that the advancement of melanoma treatment has led to an increase in the incidence of LMM because the CSF serves as a sanctuary for cancer cells given that it is a protected environment from systemic therapy [73,76].

Melanoma is one of the most aggressive forms of skin cancer and the third most common skin cancer after basal and squamous cell carcinomas. As the name implies, melanoma derives from the pigment-producing melanocyte basal cells of the epidermis [73,77]. This deadly cancer can metastasize from a relatively small primary tumor of less than 1 mm thickness and accounts for the greatest number of fatalities from skin cancer. It has a high rate of metastases, often to brain parenchyma and CSF. Its ability to metastasize more efficiently than other cancers arises from its superior ability to evade host defenses, genetic variability, and similarity to angiogenic cells of the vasculature [77].

As melanoma develops, it accumulates mutations that aid in its dissemination to the leptomeninges. These mutations include cell adhesion molecules like those found on cells of the vascular system. This form of molecular mimicry allows metastatic melanoma cells to resist the turbulent flow of the vasculature, extravasate from the vessel, and adhere to other organs [77,78,79,80]. Melanoma cell adhesion molecules (MCAM/MUC18) are unique to metastatic melanoma and not present in normal melanocytes. This molecule is associated with extravasation, and the immunotherapies targeting it have been effective in reducing extravasation and adhesion to certain cells [77,78,79,80]. There is evidence for an association between MUC18 and matrix metalloproteinase (MMP) activity. MMPs degrade the basement membrane and allow melanoma to invade the vasculature. Other adhesion molecules that aid in the dissemination of melanoma include L1-CAM, E-cadherin, N-cadherin, and many more [77]. Additionally, metastatic melanoma often harbors a mutation in the BRAF gene. This mutation causes an upregulation of the MAPK pathway, which is associated with growth and proliferation [72].

Metastatic melanoma cells resist the turbulent flow of the vasculature and migrate to the vessels of the arachnoid mater and pia mater [77]. Once they reach this site, the metastatic melanoma can extravasate from the vasculature to the subarachnoid space, where it spreads through the CSF [77,78,79,80]. Once the melanoma has extravasated, it must establish a blood supply through angiogenesis to proliferate. It does so by expressing certain inflammatory molecules like IL-8 and vascular endothelial growth factor A (VEGFA), which is induced by IL-8 [77,78,79,80].

Patients with primary melanoma can be cured, but metastatic melanoma patients have a 5-year survival rate of less than ten percent. For this reason, early diagnosis of melanoma is vital [77]. The diagnosis of LMM can be impeded by the diverse and non-specific presentation of headaches, pain, weakness, and neurological deterioration [73]. Similar to other LMDs, T1-weighted magnetic resonance imaging (MRI) with gadolinium contrast and cytology are the gold standard diagnostic tools of choice for LMM [3,73]. Cytology is more definitive for diagnosing LMM as it has high specificity but low sensitivity [73].

Melanocytes originate from neural crest cells during development, and these cells can develop into rare primary CNS melanomas. Primary CNS melanomas can be differentiated from metastatic melanoma based on clinical presentation. Primary CNS melanomas usually develop in younger patients under 50 years old and most often metastasize to systemic organs. If a systemic focus is not found, a diagnosis of primary CNS melanoma can be made.

Other emerging diagnostic tools include ctDNA and CellSearch^®^ Veridex, which are used to detect circulating tumor cells (CTC) in the blood [81,82]. ctDNA is cell-free DNA, which is circulating DNA that arises from cells that have died. In ctDNA detection, the presence of mutations in floating DNA, like BRAF or KRAS mutations, is quantified using Next Generation Sequencing (NGS) or Droplet Digital PCR (ddPCR). In one study by Ballester et al. *(n =* 7), the researchers found a higher correlation between ddPCR and MRI than between CSF cytology and MRI. They also noted that NGS did not produce correlative results with MRI [81]. Furthermore, they concluded that NGS and ddPCR have high specificity, as they did not note any false positives. Ultimately, they concluded that ddPCR is a better diagnostic tool than CSF cytology. In the study by Le Rhun et al., they found that CellSearch^®^ Veridex could accurately detect malignant cells; however, the sample size only included two patients. More rigorous research is required to prove the efficacy of this modality [82].

Given the poor prognosis of LMM, the goal of treatment is palliative, with a focus on prolonging life while maximizing comfort and avoiding toxicities from medications [3,73]. Conventional methods of therapy include radiation therapy, the placement of an Ommaya reservoir/VP shunt to relieve hydrocephalus, and systemic therapy. The efficacy of radiation therapy is still being investigated, with variable results [3,73,83]. Patients are evaluated for responsiveness to treatment based on the Karnofsky Performance Status (KPS) scale, which measures functional capacity [3]. If they have a score less than 60, patients are less likely to be responsive to treatment, and it is recommended that they be spared from aggressive interventions [84].

Clinical evidence and the development of immunotherapy for patients with LMM are limited given the exclusion of LMM patients from many clinical trials. ICS and targeted therapies (TT) are treatments that have not been thoroughly investigated. In the past, patients would receive whole-brain radiotherapy as a palliative measure for headaches and intrathecal chemotherapy [14,73,74,85,86,87].

Patients with melanoma are identified as being BRAF + or −, which greatly impacts their treatment options. The discovery of the BRAF mutation in half of all melanoma cases and the creation of small-molecule BRAF kinase inhibitors like vemurafenib revolutionized the treatment of melanoma. It remains unclear whether the concentration of these drugs reaches therapeutic levels in the CSF. BRAF + patients with LMM have been shown to be responsive to vemurafenib, dabrafenib, and trametinib; however, evidence mostly consists of case reports, and more vigorous research is required to elucidate the efficacy of these drugs [86,87,88]. Similarly, checkpoint inhibitors like ipilimumab, nivolumab, pembrolizumab, and intrathecal interleukin-2 (IL-2) show promise in the treatment of LMM, but the evidence is limited. Several clinical studies studying the efficacy of combined drug interventions, including IT and checkpoint inhibitors, in the treatment of several tumor types, including melanoma and LMM, are in progress [73,89]. Overall, patients who received systematic treatment only had a survival rate of 3.9 months longer than those who did not [14,90].

## 5. Primary Central Nervous System Tumors

Leptomeningeal dissemination is observed in 10–20% of primary CNS tumors. Pediatric tumors result in more frequent dissemination, with 20–30% expressing positive CSF cytology [91]. >95% of primary nonhematologic CNS tumors with metastasis were high-grade or malignant [91]. Histopathological characteristics, such as histopathological tumor grade and tumor angiogenesis, have been associated with leptomeningeal spread. CSF seeding during surgery is another proposed mechanism for particular tumors. The majority of metastatic tumors in CSF displayed cytomorphology reminiscent of the primary tumor [91].

**Figure 3:**
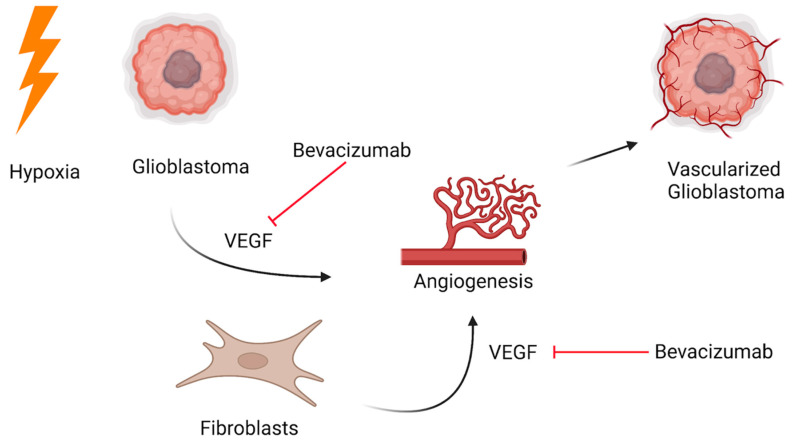
Targeted bevacizumab therapy for leptomeningeal disease originating from the central nervous system.

Management primarily consists of surgical management as a mainstay, often coupled with chemoradiotherapy. These approaches generally consist of whole craniospinal irradiation and intrathecal chemotherapy. Radiotherapy, despite its increasing use in pediatric treatment, is still associated with significant long-term consequences and is thus avoided when possible. Long-term sequelae include neurocognitive defects, endocrine dysfunction, growth defects, and an increased risk of a second malignancy. More recently, clinical trials have highlighted anti-angiogenic inhibitors as having promising therapeutic value [92]. Systemic anti-angiogenic treatment with bevacizumab alone and in combination with chemoradiotherapy translated into effective symptom control in five out of nine patients and increased overall survival (Figure 3).

**Figure 4:**
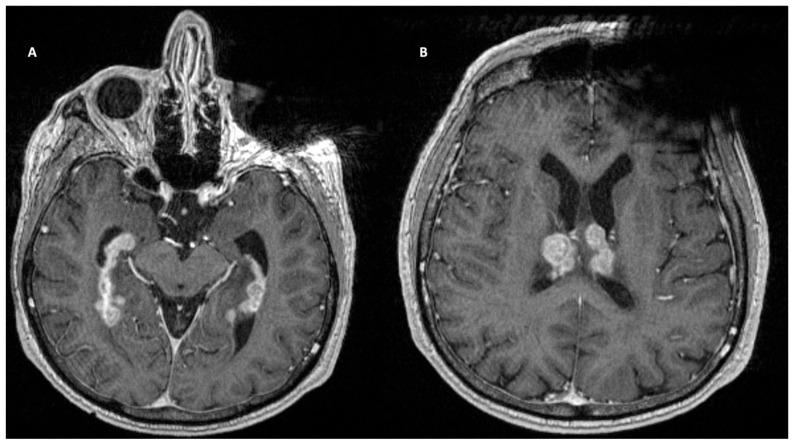
T1-weighted contrast-enhanced MRI demonstrated a large B-cell lymphoma centered in the subependymal regions of the lateral ventricles.

Glioblastoma (GBM) has been shown to metastasize to the leptomeninges. Known to spread locally throughout the brain parenchyma, malignant astrocytic tumors are only associated with leptomeningeal spread in 10–21% of cases. Demir and colleagues report the dissemination of a pediatric GBM [93]. Pohar and colleagues describe another metastatic GBM in a 63-year-old female [94]. Spread to the leptomeninges is found to be primarily hematogenous or by direct extension along compact fiber pathways, such as nerves or subependymal regions (Figure 4). In this patient, the tumor infiltrated the lateral ventricle and spread via CSF. On histopathological examination, the tumor was found to be positive for GFAP and vimentin. Studies have linked low GFAP expression with increased leptomeningeal spread [95]. This may indicate that less differentiated astrocytes are implicated in metastasis. An important molecular marker specific to GBM is isocitrate dehydrogenase (IDH). According to WHO, IDH-1 and IDH-2 mutations are found in 10–15% of GBMs. Gliomas expressing IDH-1 wild-type may be associated with leptomeningeal spread. Histone gene mutations are another well-known predictor of leptomeningeal dissemination in GBM. H3K27M mutations are associated with posterior fossa and midline tumors and are associated with leptomeningeal disease and a poor prognosis. The primary management of this tumor is surgical. Gross craniotomies and gross total resections were performed in the aforementioned studies. The standard of care generally involves combination adjunctive chemoradiotherapy. In general, the prognosis is poor, and treatment is palliative. Multiple case reports have identified the leptomeningeal spread of GBM [96,97]. A systematic review showed a median time to spinal metastasis of 10 months and a median overall survival of 3 months following spinal metastasis. Palliative laminectomies have also been performed to ameliorate symptoms. Neither palliative laminectomy, CT, nor RT have demonstrated therapeutic advantage. Interestingly, surgery may have a therapeutic advantage, as those who underwent only a biopsy had a shorter time to develop spinal metastasis [98].

Germ cell tumors also account for a share of leptomeningeal metastases. Intracranial germinomas account for 3% of neoplasms in children aged 0–19 in North America and 15% of intracranial neoplasms in Asia. Malignant germinomas spread in a periventricular pattern through infiltration of the subependymal lining. Classical biomarkers include an elevated alpha-fetoprotein and a low-to-normal beta human chorionic gonadotropin. Due to the prevalence of variants, these biomarkers can vary, making diagnosis challenging. Historically, these have been treated with craniospinal irradiation. Due to high levels of toxicity, this approach is being replaced by targeted local and ventricular approaches. Preradiation chemotherapy is also being utilized to allow for lower doses of radiation to be administered.

Medulloblastomas and neuroendocrine tumors are also associated with drop metastasis. In a case study on medulloblastoma, the patient was positive for synaptophysin and chromogranin A and negative for GFAP, EMA, and CD20 [99]. Genetic analyses have identified key genes involved in metastasis. Overexpression of Arnt and Gdi2 genes in sonic hedgehog-induced medulloblastomas was found to increase migration, invasiveness, and anchorage-independent growth, increasing the metastatic compliance of cells. MiRNA-21 and ID3 have also been implicated in metastasis, and their suppression has been associated with improved outcomes [100,101,102]. One survival analysis found late-drop metastasis to be a negative prognostic factor, revealing a significant drop in overall survival. In this population, although palliative, treatment of leptomeningeal metastasis was found to stabilize and protect the brain from further neurologic deterioration. Patients in high-risk subgroups received adjuvant craniospinal radiation and intrathecal chemotherapy, as well as stereotactic radiosurgery. Detection of this metastasis was also found to be a significant challenge, with advanced neuroimaging needed to diagnose it [103]. Pinealoblastoma is another rare neuroendocrine neoplasm with documented spinal drop metastasis. These occur in the pineal region and contain highly anaplastic and necrotic features, as well as rosette structures. Additionally, they are positive for neural markers, such as synaptophysin and negative for glial markers, such as GFAP, pancytokeratin (PanCK), and epithelial membrane antigen (EMA). Initially, pineoblastomas are managed with maximal tumor resection coupled with adjuvant chemoradiotherapy. The presence of leptomeningeal metastasis is an indicator of a poor prognosis. Pinealoblastomas are theorized to hematogenously seed in the spinal fluid. Inactivation of Rb and P53 has been associated with leptomeningeal metastasis of pinealoblastoma. Surgical management of metastatic disease is a mainstay for this pathology, followed by adjuvant chemoradiotherapy [104]. Another case study identified a primary neuroendocrine tumor of the pineal gland with drop metastasis to the spine [105]. Choroid plexus carcinoma is a rare intraventricular neoplasm affecting children. The tumor’s site of origin, the choroid plexus epithelium, makes it prone to seeding the neuraxis through cerebrospinal fluid. Maximal surgical resection coupled with adjuvant chemoradiotherapy is the current preferred treatment regimen. Radiotherapy, namely craniospinal irradiation and whole brain radiotherapy, has been identified as providing better clinical outcomes than chemotherapy. Despite this, chemotherapeutic approaches are under investigation, although an optimal approach is currently unknown.

Embryonal tumors are highly malignant neoplasms primarily affecting young children with a tendency to metastasize along the neuraxis. Embryonal tumors with abundant neuropil and true rosettes (ETANTR) are a set of embryonal tumors with a poor prognosis. Unique molecular features include miRNA cluster C19MC amplification and LIN28A positivity. These features are associated with aggressive tumor growth, tumorigenesis, and poor survival. Optimal treatment for this tumor involves maximally safe surgical resection and adjuvant risk-adapted chemoradiotherapy. Proton therapy is a promising frontier in radiotherapy that can limit damage to the heart, lungs, and bowel in patients with ETANTR. Atypical teratoid/rhabdoid tumor (AT/RT), a rare embryonal CNS neoplasm, is known to metastasize to the leptomeninges. Tumor dissemination is reported in 24% of patients at the time of diagnosis and in another 35% within 3 years. Metastatic AT/RTs appear as large cells with rhabdoid morphology. AT/RTs are characteristically INI1-negative due to a SMARCB1 gene deletion. The standard of care for metastatic disease favors chemotherapy to avoid the toxic effects of radiotherapy. Despite this, some studies suggest that aggressive early craniospinal irradiation in children > 3 years of age is imperative to prevent further metastasis. Intrathecal chemotherapy has been explored as a means of treating metastatic AT/RT as an alternative to radiation. In these trials, various combinations of methotrexate and cytarabine have demonstrated success.

**Figure 5:**
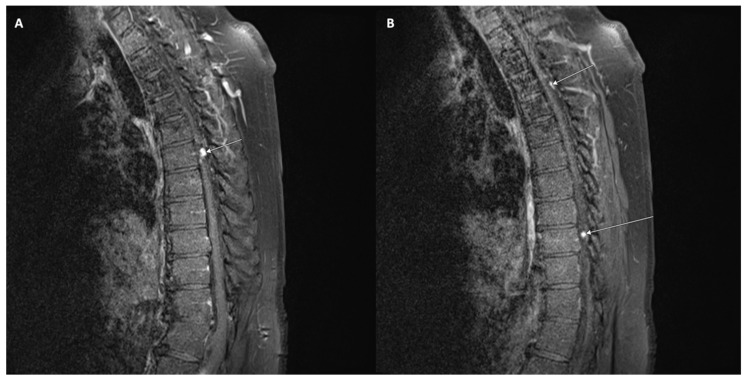
T1-weighted contrast-enhanced MRI demonstrating drop metastases at T6 (**A**), T3, and T9 (**B**) in a patient who previously underwent a resection for an ependymoma.

Spinal seeding metastasis has also been observed in oligodendrogliomas. These tumors are associated with recurrence and lethal outcomes. Drop metastasis is seen in 1–2% of all cases. This phenomenon is depicted in Figure 5. There is no information on how this occurs, and blood and lymphatic metastasis are proposed mechanisms. The time to metastasis has been reported to be anywhere between 3 months and 6 years. Metastasis has been associated with a higher histopathological grade, although seeding can occur without malignant transformation. Following decompression surgery, this patient was managed with chemoradiotherapy [106]. Another case report describes a similar patient with spinal metastasis following resection of a low-grade frontal oligodendroglioma. In this case, surgical resection allowed for an overall survival of 1 year after the initial diagnosis. Spinal metastasis was found to occur years after resection of the primary tumor in this case, and spinal seeding was demonstrated to be a negative event indicating short survival [107].

Primary CNS melanoma has also been associated with metastasis. Melanoblasts are of neural crest origin and migrate to the skin, uvea, mucous membranes, and leptomeninges during development. Leptomeningeal spread may be linked to its histogenetic origin [108]. Somatic mutations most commonly occur at the Q209 and R183 residues of GNAQ and induce tumorigenesis through upregulation of the MAP kinase pathway. Primary CNS melanoma either invades the meninges diffusely or presents as nodular intraparenchymal lesions. The ease of meningeal invasion may be because it is highly vascularized. The prognosis for metastatic disease is very poor. As such, the treatment approach is primarily palliative, not curative. Treatment included radiotherapy and intra-CSF chemotherapy. Survival time in this case was 13 months after diagnosis. Another case report identified a primary temporal lobe malignant melanoma with extracranial metastasis to the spinal cord and lungs [109].

Meningioma has also been associated with leptomeningeal spread. A case report described a 27-year-old female with rhabdoid meningioma. Histological examination revealed rhabdoid and papillary meningiomas, which were vimentin and S-100 positive with EMA expression. The patient was managed with a subtotal resection followed by radiotherapy. The patient died 3 months after symptom onset. It is believed the combination of anaplasia, high mitotic index, and loss of cohesion between neoplastic cells led to diffuse leptomeningeal metastasis [110].

## 6. Hematologic Cancers

Although hematologic malignancies range in subtypes, they refer to cancers afflicting the blood, bone marrow, and lymph nodes [111]. This grouping includes cancers, such as lymphoma, leukemia, and multiple myeloma. Lymphomas are categorized as either Hodgkin’s lymphoma or NHL [112]. Hodgkin’s lymphoma (10% of lymphoma cases) is marked by the presence of Reed-Sternberg cells and CD15 and CD30 staining, along with distinct clinical characteristics, whereas NHL (90% of lymphoma cases) lacks these specific features [113]. Rather, NHL is characterized by CD19- and CD20-positive markers [114]. Additionally, Hodgkin’s lymphoma and NHL can further be subdivided into over 90 different subtypes [115]. Leukemias consist of cancer subtypes, such as acute myeloid leukemia, chronic myelogenous leukemia (CML), ALL, and chronic lymphoblastic leukemia (CLL) [116]. With respect to LMD spreading as a late-stage complication to systemic cancers, lymphoma (specifically NHL), leukemia (ALL), and multiple myeloma comprise some of the common primary etiologies in addition to some of the solid tumor cancers as detailed in previous sections [1].

Regarding incidence, NHL is the most common hematologic malignancy, contributing to around 4% of cancer diagnoses in the United States, with about 77,000 reported cases in 2020. Globally, NHL diagnoses reached closer to 500,000 cases in 2018. It is estimated that the NHL was responsible for around 250,000 deaths worldwide in 2018. This value comprises 2.6% of annual mortalities attributable to cancer. In the United States, the 5-year survival rate for NHL-diagnosed individuals was 72.7% (83.5% stage I, 63.3% stage IV) between 2010 and 2016. The average diagnosis age is around 67 years old [117]. Lymphomagenesis, or the development of lymphoma, is the result of complex genetic and environmental interactions, including lymphocyte signaling, transcription factor regulation, and apoptotic mechanisms that synergistically result in the transformation of T and B lymphocyte cells [118]. Tumorigeneses consist of uncontrolled lymphocyte proliferation associated with distorted signaling pathways [119]. Similar to other cancer types, the gold standard for a likely lymphoma diagnosis is an excisional biopsy at the level of the lymph node [115]. Although fine needle aspiration or partial biopsy present possible less invasive alternatives to open excisional biopsy, the former procedures neither provide the same level of sensitivity nor specificity. Moreover, extraction of adequate nodal structures provides not only improved diagnosis but also benefits staging assessment and management planning [120]. Imaging modalities for early lymphoma detection and subsequent interim and remission monitoring involve a combination of CT and [18F]FDG-PET scans [121]. The PET-CT protocol has increasingly become the standard of care for lymphoma patients as it provides both an anatomical and functional frame of reference [122]. However, the frequency and duration of interim and remission imaging conventions are less established. The benefit versus cost of periodic imaging is unclear given differing result-based guidelines [123]. Staging and grading the lymphoma case adds another factor to consider, as this will play a role in the rate of lymphoma progression and whether or not these changes are detectable under image guidance.

Of note, diffusion-weighted MRI is particularly useful in the detection of primary CNS lymphoma. Primary leptomeningeal lymphoma comprises a small subset of primary CNS lymphomas. Leptomeningeal involvement in lymphoma is exceedingly rare, only occurring in 6% to 8% of NHL patients. However, the prevalence of LMD in NHL accounts for 5 to 30% of all LMD metastatic cases, with a median prognosis of 2.6 months of survival [1]. Consequently, LMD metastasis presents a poor prognosis, with an overall survival estimated at less than 6 months in duration [124]. Given that specific lymphoma variants have relatively higher rates of CSF dissemination, CSF cytology remains a crucial diagnostic tool for leptomeningeal lymphoma [125]. Unfortunately, multimodal diagnostics in the form of CSF analysis and focal MRI detect leptomeningeal involvement in 7% to 42% of patients, thereby indicating observable variation in accuracy [124]. Compared to solid tumor cancers, LMD from lymphoma occurs more often in the absence of systemic disease or parenchymal involvement and, at times, during remission.

Lymphoma-specific cell markers are numerous secondary to the quantity of subtypes possible. B-cell markers include CD20, Pax-5, CD79a, Oct-2, and BOB.1. Hodgkin lymphoma markers include CD15, CD30, and CD57. T/NK-cell markers include CD2, CD5, and CD4. The absence of both CD4 and CD8 is a hallmark of T-cell lymphoma [126]. Standard of care treatment for lymphoma patients is multimodal and comprises both chemotherapy and radiotherapy in addition to antibody-drug conjugates (immunotherapy) [119]. Since 2018, brentuximab vedotin, an anti-CD30 antibody-drug conjugate, has been FDA approved for certain lymphoma subsets. Brentuximab vedotin has proven to be efficacious in treatment and has a clinically safe toxicity threshold [120]. Rituximab, which targets CD20, is a relatively more established antibody treatment against NHL [127]. As previously stated, CSF analysis is a prioritized diagnostic mode in dealing with primary central nervous system lymphomas. More specifically, microRNA profiles have been demonstrated to be accurate in detecting primary central nervous system lymphoma in addition to other CNS/neurological disorders. Some of these markers include miR-21, miR-19, and miR-92a. Subsequent miRNAs have been shown to result in 95.7% sensitivity and 96.7% specificity [128]. Additionally, activated nuclear factor kappa-B is another marker highly reminiscent of primary central nervous system lymphoma. Current clinical trials have aimed to examine the underlying mechanisms behind the efficacy of Bruton’s TKIs in the management of this lymphoma subset [129]. Overall, the use of these noninvasive biomarkers in conjunction with imaging monitoring and appropriate treatment has provided improved outcomes in recent years.

With respect to leukemia, the Global Cancer Observatory (GCO) observed a global incidence of around 474,000 cases. ALL and AML have a probable likelihood of manifesting in both children and adults, reflecting a bimodal age distribution. Conversely, CML and CLL are more commonly diagnosed in older populations. There were an estimated 61,000 newly diagnosed cases of leukemia in 2021 based on the Surveillance, Epidemiology, and End Results (SEER) database, contributing 3.2% of all cancer diagnoses. The estimated number of deaths associated with leukemia amounted to 23,660 known deaths, which contributes to 3.9% of all cancer-related deaths [130]. Suspected leukemia can first be detected through an abnormal complete blood count (CBC), specifically in the form of leukocytosis or an elevated white blood cell count (WBC) [131]. Further suspicions of leukemia are confirmed through additional blood testing, flow cytometry analysis, and bone marrow sampling. Imaging in the form of CT/MRI is more commonly employed for CNS diseases involving leukemia. MRI (conventional, post-contrast, MR venography, and diffusion-weighted MRI) is particularly critical to the early detection of CNS lesions, which are often treatable [132]. Since imaging abnormalities indicative of CNS involvement in leukemia vary, radiologic findings have to be corroborated with lab work and symptoms for a timely diagnosis. Regarding biomarkers, CNS-associated leukemia is accompanied by elevated expression of adhesion molecules from these leukemic cells, including VLA-4, ICAM-1, VCAM, L-selectin, PECAM-1, CD18, LFA-1, CD58, CD44, and CXCL12 [133]. These leukemic cells also secrete VEGF-A, resulting in disruption of the BBB and infiltration into the CSF. As a result, CSF cytology remains a necessary component in the diagnostic workup of lymphoma with CNS extension. Biopsy and/or bone marrow aspiration are another relatively more invasive gold standard in the early detection of lymphoma of all subtypes. Treatment options will usually entail chemotherapy, with radiotherapy more often employed to prevent metastasis or treat CNS infiltration [131]. Other treatment options include hematopoietic stem cell transplantation (typically allogenic bone marrow transplantation) and monoclonal antibodies [134]. However, high-dose chemotherapy and stem cell transplantation are less suitable for older, comorbid populations. Hypomethylating agents (HMAs) and medications, such as venetoclax (a BCL-2 apoptotic regular protein inhibitor), are promising new noninvasive additions to current treatment regimens [135,136]. Furthermore, FMS-like tyrosine kinase 3 (FLT3) and isocitrate dehydrogenase inhibitors have been proven to work as successful therapies alone and in conjunction with other modalities in a percentage of lymphoma patients. Unfortunately, these developing treatments also present their own shortcomings, such as mechanisms of resistance to said medications [137].

Behind NHL, multiple myeloma ranks as the next most common hematologic cancer type. However, multiple myeloma only comprises 1% of all cancer cases. Multiple myeloma is characterized by abnormalities in plasma cells within the bone marrow, resulting in uncontrolled proliferation [138]. It is estimated there were around 24,000 to 30,000 annual cases, accompanied by 12,650 deaths, in 2016. Similar to lymphoma, the median age at diagnosis of multiple myeloma has been calculated to be 66 to 70 years old. Across multiple hematologic and solid tumor cancers, survival rates have been trending towards extended periods of survival given the increased availability of effective therapies. Median survival in multiple myeloma patients has approximately doubled in duration from 12 months prior to the year 2000 to 24 months after 2000 [139].

The diagnosis of multiple myeloma results from a summation of a history/physical, lab work including urinalysis and bone marrow biopsy, and radiography [140]. The extent of organ damage dictates the urgency and severity of treatment. Organ damage indicates the urgency of treatment. Along with organ damage, bone disease is a common multiple myeloma feature, whereby skeletal radiography is necessary for detection [141]. Previous conventional radiography consisted of a generalized skeletal x-ray series. However, as this procedure alone lacks the sensitivity of osteolytic lesion detection, radiographic protocols have progressed to multiple, more sophisticated imaging modalities, including whole-body low-dose CT, whole-body MRI, and PET/CT [142]. These imaging techniques range in time, sensitivity, and cost; they should be used both together and under context specific criteria. The standard of care for newly developed multiple myeloma consists of triplet therapy along with high-dose chemotherapy and autologous stem cell transplantation [143]. Triplet therapy refers to treatment with a corticosteroid, an immunomodulator, and a cancer targeting drug, such as proteasome inhibitors [144]. The use of monoclonal antibodies, such as daratumumab and elotuzumab, which target CD38 and SLAMF7 markers, has been effectively integrated into clinical treatment [145]. However, the same issue is present across multiple antibody treatments, where clonal selection for resistance in multiple myeloma cells arises. B-cell maturation antigen (BCMA)-targeted therapies are potentially new alternatives that have demonstrated efficacy without excessive toxicity [146]. LMD metastasis/CNS involvement cases from primary multiple myeloma are not frequent and are often evidenced through case reports. Medication penetration through the BBB is a consistent obstacle once malignancies metastasize to the CNS. Novel therapies are currently being studied in both preclinical and clinical settings to overcome this barrier. Thalidomide and pomalidomide have proven to show adequate penetration. Moreover, marizomib is a proteasome inhibitor that also demonstrates similar penetrative qualities [147]. As stated, LMD metastasis from hematologic cancer is a relatively rare scenario. The lack of studies regarding appropriate treatment, dosing, and overall approach to LMD highlights a demand for further study in this context.

## 7. Leptomeningeal Mimics

Thus far, this review has elucidated the pathophysiology and therapies of the most common forms of LMD, with a closing discourse on hematologic origins. Therefore, a brief discussion of rarer yet equally intriguing presentations of LMD will also be introduced. To note, leptomeningeal involvement need not be attributed to metastatic or malignant etiologies. Various diseases may present with leptomeningeal involvement, mimicking LMD. Currently, these have been sparsely described in the literature, primarily in the context of case reports, including rare instances of neuroborreliosis, giant cell arteritis, and sarcoidosis [148,149,150].

In particular, sarcoidosis appears to be the most widely encountered mimic of LMD in instances of CNS involvement. Sarcoidosis is well acknowledged as a systemic ailment characterized most aptly upon histological observation of non-caseating sarcoid granulomas [151]. Its prevalence ranges from 0.001% to 0.04% of individuals, more widely affecting females and those aged 20–40 [152,153]. To date, it carries an unexplainable etiology, shifting considerable diagnostic reliance on biopsy. The majority of sarcoidosis cases affect the respiratory system in nearly 90% of individuals, inadvertently lowering suspicion in the context of other organ involvement [151]. However, in the context of CNS involvement, it is particularly salient to acknowledge any form of systemic involvement prior to the onset of neurological symptoms. This can provide considerable guidance towards the diagnosis of sarcoidosis pertinent to the regions of the CNS—neurosarcoidosis [152,154].

Neurosarcoidosis can affect the brain, cranial nerves, meninges, and spinal cord [154]. Symptomatic presentation can manifest as generalized neurological symptoms including headache, diplopia, and vertigo, among others, providing low utility regarding neurosarcoidosis diagnosis, though the sequence of region involvement can provide more value in this; moreover, the number of systems involved has displayed a notable association [154]. For example, in a prospective study of 166 patients, systemic manifestation in 100 cases coincided with, in 55 cases preceded, and in only 10 cases followed CNS involvement chronologically [154]. Other pertinent biomarkers included MRI and CSF abnormalities, as well as LMD. This involved hyperproteinorrachia, hypoglycorrhachia, and elevated angiotensin converting enzyme (ACE) CSF levels [154]. MRI observations included regional enhancement on T1-weighted MRI scans following gadolinium administration [155]. Overall, it veers toward a rule-out approach, requiring sound exclusion for other etiologic explanations. Current treatment options are rarely definitive and exist primarily in the form of inflammatory control, as with other forms of sarcoidosis, including glucocorticoids and inflammatory modulators, such as tumor necrosis factor (TNF) alpha inhibitors [156].

Rarer instances of LMD mimics are relatively sparse, though these will be discussed. As elucidated, neuroborreliosis has been accounted for in the literature as a potential condition highly reminiscent of leptomeningeal involvement. Neuroborreliosis entails the neurological affiliation of infection by the genus Borrelia, colloquially acknowledged as Lyme disease. Such affiliation following CNS involvement occurs in up to 15% of borrelia infections, often manifesting as non-specific neurological symptoms including meningitis, cranial nerve deficits, and radiculopathy. The gold standard of diagnosis for LMD, MRI, has little distinctive capacity between neuroborreliosis and LMD, thereby producing complex cases in the current literature. Caretakers should then be highly suspicious of the former in the setting of potential signs of vector-borne infections, such as erythema and rash, as reported previously. Upon suspicion, further serological testing via antibody detection of borrelia has more definitively ruled out a diagnosis of neuroborreliosis. Treatment intuitively entails antibiotic therapy, which exclusively resolves neuroborreliosis as opposed to LMD.

Giant cell arteritis has likewise been scantily reported, though it has appeared as a potential mimic of LMD. Giant cell arteritis (GCA), synonymously termed temporal arteritis, is a vasculitis affecting medium- and large-sized vessels, quite often the external carotid artery and its branches, namely the temporal branch. A primary concern in GCA is that of ischemic optic neuropathy, which is understandably observed in the form of vision loss. Indeed, the case report detailing the leptomeningeal mimicking characteristics of giant cell arteritis was characterized by a similar presentation with progressive vision loss. Additionally, MRI may reveal nodular enhancement, more so localized to the optic nerve sheaths, which is also reminiscent of sarcoidosis. Temporal artery biopsy is the most conclusive modality for diagnosing GCA, though the path towards this diagnosis remains relatively difficult to land upon. Similar to sarcoidosis and other inflammatory disorders, steroid treatment has demonstrated efficacy.

## 8. Conclusions

Although LMD metastasis from both solid tumor cancers and hematologic malignancies is comparatively more frequently occurring than primary diffuse leptomeningeal gliomatosis, it still presents as a late-stage complication in these cancer types in a small subset of metastatic cancer patients (approximately 5%), with diagnosis often occurring by way of post-mortem autopsy [157,158]. The overall prevalence of LMD accounts for approximately less than 10% of diagnosed cancer cases [50]. Interestingly, LMD occurrence has been more frequent in recent years due to the extended outcome survival for cancer patients as a whole [1]. Regardless of primary etiology, early detection and treatment of CNS involvement have proven to provide relatively improved outcomes, even if still measured at a median of 3 to 6 months in duration. Survival periods are not long by nature, but they have lengthened as the overall number of cases of LMD has increased. This trend is in part due to the advances in detection strategies that utilize a combination of MRI, CT, and CSF cytopathology by way of lumbar puncture analysis. Imaging is not without limitations, however, as MRI detection has a rated sensitivity and specificity of 75% and 77%, respectively [158]. Additionally, CSF samples require more than one round. CSF analysis remains the gold standard and is able to mediate unclear imaging enhancements. In recent advances, genomic analysis of ctDNA has shown itself to be a promising biomarker for neurological malignancies [159]. Treatment for LMD has likewise progressed from more systemic forms to targeted therapy based on the primary cancer types. Utilizing inhibitors against cancer-specific markers overcomes the limitations of widespread chemotherapy, which fails to fully pass the BBB [160]. Improved therapy for LMD requires progress in targeting specific cancer types. As stated, LMD metastasis is a late-stage complication of several different primary cancers. Prognosis is not optimal as LMD diagnosis may reflect failed therapy to control metastasis; however, medical advancements are trending towards more accurate and salient forms of detection and treatment of not only metastatic malignancies but also CNS-specific malignancies, such as LMD.

## Data Availability

No datasets were utilized.

## Abbreviations

LMD	Leptomeningeal disease
MS	Multiple sclerosis
CNS	Central nervous system
NHL	Non-Hodgkin’s lymphoma
ALL	Acute lymphoblastic leukemia
CSF	Cerebrospinal fluid
CN	Cranial nerve
NCNN	National Comprehensive Cancer Network
KPS	Karnofsky performance scale
SWS	Sturge-Weber syndrome
MRI	Magnetic resonance imaging
CEA	Carcinoembryonic antigen
ICP	Intracranial pressure
VPS	Ventriculoperitoneal shunt
WBRT	Whole brain radiation therapy
CSI	Craniospinal irradiation
ICI	Immune checkpoint inhibitor
RCT	Randomized controlled trial
MC	Meningeal carcinomatosis
LBM	Leptomeningeal breast cancer metastasis
ER	Estrogen receptor
PR	Progesterone receptor
HER2	Human epidermal growth factor receptor 2
EGFR	Epidermal growth factor receptor
BBB	Blood brain barrier
ctDNA	Tumor derived DNA
HCB	Hot cross bun
LLM	Leptomeningeal lung cancer metastasis
NSCLC	Non-small cell lung cancer
BM	Brain metastases
ALK	Anaplastic lymphoma kinase
SCLC	Small cell lung carcinoma
LUAD	Lung adenocarcinoma
LUSC	Lung squamous cell carcinoma
ACTA2	Actin gene
EpCAMs	Epithelial cell adhesion molecules
TKI	Tyrosine kinase inhibitor
LMM	leptomeningeal melanoma metastasis
MCAM	Melanoma cell adhesion molecule
MMP	Metalloproteinase
VEGFA	Vascular endothelial growth factor A
CTC	Circulating tumor cells
NGS	Next Generation Sequencing
ddPCR	Droplet digital PCR
TT	Targeted therapies
GBM	Glioblastoma
CML	Chronic myelogenous leukemia
CLL	Chronic lymphoblastic leukemia
GCO	Global cancer observatory
SEER	Surveillance, epidemiology, and end results
CBC	Complete blood count
WBC	White blood count
HMA	Hypomethylating agent
FLT3	FMS-like tyrosine kinase 3
BCMA	B-cell maturation antigen
ACE	Angiotensin converting enzyme
